# Supplementary material for: The Association between Anemia and Parkinson’s Disease: A Nested Case-Control Study Using a National Health Screening Cohort
Source: Brain Sci. 2021 May 13;11(5):623. doi: 10.3390/brainsci11050623 (PMC8152756; doi:10.3390/brainsci11050623)
Supplement: Supplementary file 1 [file brainsci-11-00623-s001.zip › brainsci-1174687-supplementary.pdf]

Table S1. Subgroup analyses of the unadjusted and adjusted odds ratios (95% confidence intervals) for anemia in the Parkinson's disease and control groups according to obesity, smoking, alcohol consumption, blood pressure, and fasting blood glucose

**Characteristics**	**Odds ratios for anemia**			
	**Unadjusted**	***p* value**	**Adjusted[b]**	***p* value**
BMI <23 (n = 11,747)				
PD	1.05 (0.95-1.17)	0.342	1.03 (0.92-1.15)	0.618
Control	1.00		1.00	
BMI ≥23 (n =17,473)				
PD	1.22 (1.10-1.35)	<0.001[a]	1.15 (1.06-1.28)	0.001[a]
Control	1.00		1.00	
Nonsmoker (n = 22,178)				
PD	1.10 (1.01-1.19)	0.024[a]	1.08 (0.99-1.18)	0.070
Control	1.00		1.00	
Past smoker and current smoker (n = 7,042)				
PD	1.21 (1.02-1.42)	0.029[a]	1.13 (0.99-1.34)	0.190
Control	1.00		1.00	
Alcohol consumption <1 time a week (n = 21,501)				
PD	1.10 (1.01-1.19)	0.026[a]	1.08 (0.99-1.17)	0.089
Control	1		1	
Alcohol consumption ≥1 time a week (n = 7,719)				
PD	1.14 (0.96-1.36)	0.132	1.12 (0.94-1.34)	0.219
Control	1		1	
Systolic blood pressure <140 mmHg and diastolic blood pressure <90 mmHg (n = 19,651)				
PD	1.12 (1.03-1.23)	0.048[a]	1.08 (0.98-1.18)	0.116
Control	1		1	
Systolic blood pressure ≥140 mmHg or diastolic blood pressure ≥90 mmHg (n = 9,569)				
PD	1.15 (1.00-1.31)	0.010[a]	1.12 (0.97-1.28)	0.125
Control	1		1	
Fasting blood glucose <100 mg/dL (n = 16,820)				
PD	1.08 (0.98-1.19)	0.141	1.04 (0.94-1.16)	0.441
Control	1		1	
Fasting blood glucose ≥100 mg/dL (n = 12,400)				
PD	1.20 (1.08-1.34)	0.001[a]	1.15 (1.02-1.29)	0.020[a]
Control	1		1	

Note: CCI-Charlson comorbidity index; PD-Parkinson's disease. [a] Logistic regression model. Significance at p <0.05.
[b] Models adjusted for age, sex, income, region of residence, obesity, smoking, alcohol consumption, other degenerative diseases of the nervous system, head trauma history, systolic blood pressure, diastolic blood pressure, fasting blood glucose, and CCI scores.
